# Assessing the merits: an opinion on the effectiveness of simulation techniques in tumor subclonal reconstruction

**DOI:** 10.1093/bioadv/vbae094

**Published:** 2024-06-26

**Authors:** Jiaying Lai, Yi Yang, Yunzhou Liu, Robert B Scharpf, Rachel Karchin

**Affiliations:** Institute for Computational Medicine, Johns Hopkins University, Baltimore, MD 21218, United States; Institute for Computational Medicine, Johns Hopkins University, Baltimore, MD 21218, United States; Institute for Computational Medicine, Johns Hopkins University, Baltimore, MD 21218, United States; Sidney Kimmel Comprehensive Cancer Center, Johns Hopkins University School of Medicine, Baltimore, MD 21231, United States; Department of Oncology, Johns Hopkins Medical Institutions, Baltimore, MD 21231, United States; Institute for Computational Medicine, Johns Hopkins University, Baltimore, MD 21218, United States; Sidney Kimmel Comprehensive Cancer Center, Johns Hopkins University School of Medicine, Baltimore, MD 21231, United States; Department of Oncology, Johns Hopkins Medical Institutions, Baltimore, MD 21231, United States; Department of Biomedical Engineering, Johns Hopkins University, Baltimore, MD 21218, United States

## Abstract

**Summary:**

Neoplastic tumors originate from a single cell, and their evolution can be traced through lineages characterized by mutations, copy number alterations, and structural variants. These lineages are reconstructed and mapped onto evolutionary trees with algorithmic approaches. However, without ground truth benchmark sets, the validity of an algorithm remains uncertain, limiting potential clinical applicability. With a growing number of algorithms available, there is urgent need for standardized benchmark sets to evaluate their merits. Benchmark sets rely on *in silico* simulations of tumor sequence, but there are no accepted standards for simulation tools, presenting a major obstacle to progress in this field.

**Availability and implementation:**

All analysis done in the paper was based on publicly available data from the publication of each accessed tool.

## 1 Introduction

**Figure 1.**
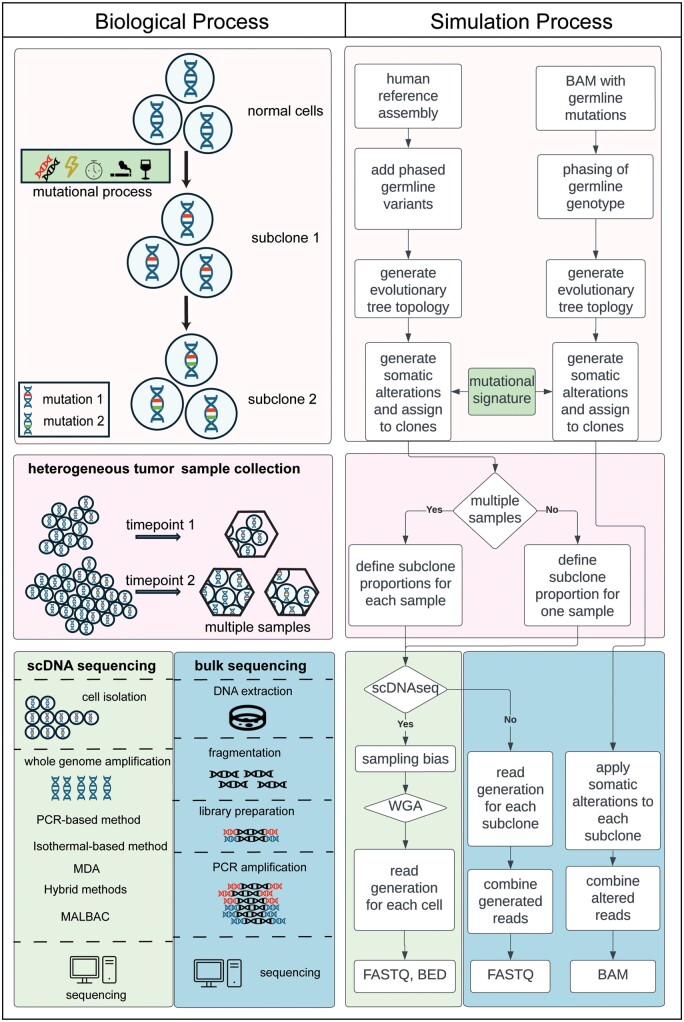
Simulation methods for tumor subclonal reconstruction are designed to mimic biological tumor evolution. Left panel: The process of tumor evolution begins when a normal diploid cell acquires somatic alterations through various mutational processes and transforms into a neoplastic cell. Through repeated mitosis, the originating cell generates a clone of genetically identical cells and continued somatic alterations result in multiple subclones which proliferate or die at different rates. The evolution of (sub)clones is represented as a tree topology, with each (sub)clone assigned to a node. Tumors sampled at different time points or spatial locations may have varying subclone compositions. Bulk DNA sequencing of tumor tissues requires DNA extraction and fragmentation, followed by library preparation in which adaptor sequences are ligated onto the fragments. For most technologies, the DNA is PCR amplified prior to sequencing. For scDNAseq, after cell isolation, most methods use whole-genome amplification to increase the amount of input material for sequencing, such as DOP-PCR, MDA, or MALBAC. Right panel: *In silico* simulation of tumor evolution begins with either a human reference assembly sequence or a germline BAM file, after which germline variants are added and/or phased to ensure a biologically realistic genome. Next, a tree topology is generated followed by simulated somatic alterations, including mutational signatures, which are assigned to (sub)clones on the tree. Each (sub)clone is assigned a proportion of the neoplastic cells in each sample. For simulation of bulk DNA sequencing, reads are generated for each subclone, then mixed to produce an output FASTQ file. For scDNAseq, reads are generated from each cell's simulated genome, and the simulated reads may correct for sampling bias or common single-cell sequencing errors, such as those resulting from WGA. The final output is either a FASTQ, BED, or BAM file for each simulated cell. SSM, small somatic mutation; CNA, copy number alteration; SV, structural variant; DOP-PCR, degenerate oligonucleotide-primed PCR; MDA, multi-displacement amplification; MALBAC, multiple annealing and looping based amplification cycles; WGA, whole-genome amplification; FASTQ, a text-based format to store raw reads; BED, browser-extensible data format; BAM, binary alignment map.

Tumors develop from a sequential selection of cellular subpopulations from an initial progenitor cell (Nowell 1976, Black and McGranahan 2021). The genetics of clonal evolution has been the subject of a large body of research, which has revolutionized our understanding of cancer. A variety of computational algorithms have been developed to reconstruct and model the hierarchical clonal architectures of tumors (Oesper *et al.* 2013, Strino *et al.* 2013, Fischer *et al.* 2014, Hajirasouliha *et al.* 2014, Jiao *et al.* 2014, Roth *et al.* 2014, Deshwar *et al.* 2015, El-Kebir *et al.* 2015, Garvin *et al.* 2015, Malikic *et al.* 2015, Niknafs *et al.* 2015, Popic *et al.* 2015, El-Kebir *et al.* 2016, Jahn *et al.* 2016, Jiang *et al.* 2016, Ross and Markowetz, 2016, Roth *et al.* 2016, Salehi *et al.* 2017, Zaccaria *et al.* 2017, Zafar *et al.* 2017, 2019, Deveau *et al.* 2018, El-Kebir 2018, Singer *et al.* 2018, Malikic *et al.* 2019a, 2019b, Borgsmüller *et al.* 2020, Cmero *et al.* 2020, Myers *et al.* 2020, Ricketts *et al.* 2020, Xiao *et al.* 2020, Zaccaria and Raphael 2020, Andersson *et al.* 2021, Baghaarabani *et al.* 2021, Satas *et al.* 2021, Sundermann *et al.* 2021, Vavoulis *et al.* 2021, Fu *et al.* 2022, Kang *et al.* 2022, Kozlov *et al.* 2022, Markowska *et al.* 2022, Zheng *et al.* 2022). Many algorithms are based on the analysis of next-generation (NGS) tumor DNA sequence reads generated from bulk DNA or single cells (scDNAseq). Clonal and subclonal markers derived from the reads include small somatic mutations (SSM), copy number alterations (CNA), structural variants (SV), and/or aneuploidies. With many algorithms now publicly available, the community needs datasets for benchmarking purposes. However, real tumor sequencing data does not provide ground truth about clonal evolution. Thus, simulating somatic alterations based on a known clonal structure has become a standard for evaluating subclonal reconstruction algorithms. For these simulations to be effective, they must accurately represent the complexities of actual tumor DNA (Fig. 1).

We identified two major categories of simulators: (1) self-contained simulators that were designed for general use and were the subject of an independent publication; (2) custom simulators that were developed to benchmark a single group's reconstruction algorithm. We evaluated nine self-contained simulators: BAMSurgeon, MosaicSim, PSiTE, Pysubsim-tree, SCNVsim, Pysim-sv, CellCoal, SimSCSnTree, and CNAsim (Qin *et al.* 2015, Chu *et al.* 2017, Xia *et al.* 2017, Yang *et al.* 2019, Posada 2020, Salcedo *et al.* 2020, Mallory and Nakhleh 2022, Srivatsa *et al.* 2023, Weiner and Bansal 2023) (Table 1). Surprisingly, self-contained simulators were used for benchmarking by only two out of 42 published subclonal reconstruction algorithms (Supplementary Table S1). For both the self-contained and custom simulators, we reported on their coverage of germline and somatic alteration types, models of clonal hierarchies, sequencing modalities, usability, support for more than one tumor sample or sequencing modality, and consideration of sequencing and caller errors.

**Table 1.** Self-contained simulation methods.

Methods	Genome alterations						Sequencing modalities											Tree	NMSS	DD	Reference
	SNV	Indel	CNA	SV	AP	MS	bulk DNA sequencing						scDNAseq								
							OT	SE	BQ	GC	WES	GP	OT	FN	FP	MV	SD				
BAMSurgeon	GS	GS	GS	GS	✔	✔	?	✔	✔	✔	–	–	–	–	–	–	–	UI	–	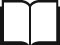	Salcedo *et al.* (2020)
Pysubsim-tree	GS	GS	GS	GS	✔	–	?	✔	✔	✔	–	–	–	–	–	–	–	UI	–	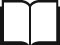	Chu *et al.* (2017)
Pysim-sv	GS	GS	GS	GS	✔	–	?	✔	✔	✔	–	–	–	–	–	–	–	BP	–	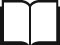	Xia *et al.* (2017)
MosaicSim	S	S	S	S	✔	✔	?	✔	–	–	✔	✔	?	–	–	–	–	CA	✔	–	Srivatsa *et al.* (2023)
PSiTE	GS	GS	S	–	✔	–	?	✔	✔	✔	✔	✔	?	–	–	–	–	CA	✔	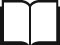	Yang *et al.* (2019)
SimSCSnTree	S	–	S	–	✔	–	?	✔	✔	✔	–	–	?	–	–	✔	–	RT	–	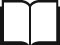	Mallory and Nakhleh (2022)
CellCoal	GS	GS	GS	–	–	✔	–	–	–	–	–	–	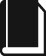	✔	✔	✔	–	CA	–	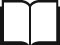	Posada (2020)
CNAsim	–	–	S	–	✔	–	–	–	–	–	–	–	?	–	–	✔	–	CA	–	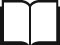	Weiner and Bansal (2023)
SCNVsim	G	G	S	S	✔	–	–	–	–	–	–	–	–	–	–	–	–	BP	–	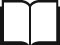	Qin *et al.* (2015)

The methods are distinguished by the types of sequence alterations they support. G, germline; S, somatic; SNV, single nucleotide variant; Indel, small insertion/deletion; CNA, copy number alteration; SV, structural variant; AP, aneuploidy; MS, mutational signature; LOH, loss of heterozygosity. Details of supported sequencing modalities are OT, output type (?, raw reads; 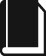, summary data such as reads counts for SNVs or segmented read depth for CNAs). Bulk sequencing features are SE, sequencing error; BQ, base quality; GC, GC bias; WES, whole-exome sequencing; GP, gene panel. scDNAseq errors are FN, false negative (such as allelic dropout and allelic imbalance); FP, false positives (such as amplification error and doublets); MV, missing value (result of non-uniform genome coverage); SD, sample distortion. The underlying evolutionary model is shown as a Tree type UI, user-input; BP, branching process; CA, coalescent; RT, random topology. NMSS, native multi-sample support = number of samples is an input to the method. DD, detailed documentation.

## 2 Few methods capture the complexity of tumor genome

Realistic tumor sequences include a mixture of reads from both tumor and normal DNA, covering various germline and somatic alterations on maternal or paternal chromosomes. Most methods initiated sequence generation with either a human reference genome FASTA file or a germline BAM file from a human subject (Fig. 1). For the former, a list of germline and/or somatic alterations was bioinformatically spiked-in to the reference sequence. The reference sequence was then used to generate reads with a dedicated package, such as ART or Wessim (Huang *et al.* 2012, Kim *et al.* 2013, Garvin *et al.* 2015, Chu *et al.* 2017, Xia *et al.* 2017, Yang *et al.* 2019, Cmero *et al.* 2020, Ricketts *et al.* 2020, Zaccaria and Raphael 2020, Mallory and Nakhleh 2022; Weiner and Bansal 2023), or was iteratively fragmented (Srivatsa *et al.* 2023). If a germline BAM file was the starting point, somatic alterations were simulated by editing the reads (Salcedo *et al.* 2020). Both approaches produced FASTQ or BAM files of simulated tumor sequence. Alternate approaches simulated alterations to the reference sequence without generating reads (Qin *et al.* 2015) or produced high-level summaries of the sequence data such as read counts for SSMs, segmented read depth for CNAs, or a cell-by-mutation matrix for scDNAseq (Table 1, Supplementary Table S1). Of the evaluated methods, only three simulation methods can handle the full range of germline and somatic alterations in tumor genomes (Chu *et al.* 2017, Xia *et al.* 2017, Salcedo *et al.* 2020) (Table 1). In addition, MosaicSim is the only tool that considers highly complex genomic alterations such as chromothripsis and kataegis, highlighting a need for more sophisticated techniques to capture the genetic diversity of tumors.

## 3 Assumptions about the process of tumor evolution vary

Simulation methods began with a model of an evolutionary process, which included a tree topology and a distribution of somatic alterations on the nodes or edges of the tree. For methods that assumed a coalescent process (Yang *et al.* 2019, Posada 2020, Srivatsa *et al.* 2023, Weiner and Bansal 2023), the topology was generated first, followed by the distribution of alterations. Alternatively, if a branching process was assumed (Popic *et al.* 2015, Qin *et al.* 2015, Xia *et al.* 2017, Ricketts *et al.* 2020, Andersson *et al.* 2021, Kang *et al.* 2022), the topology was generated in parallel with the alterations. Yet another approach was to generate a fixed or random topology and then generate the alterations, by parameterized distributions (Uniform, Poisson, Dirichlet, etc.) (Table 1, Supplementary Table S1). Four of the methods used coalescent models but did not incorporate modifications for cancer evolution, such as selective sweeps (Merlo *et al.* 2006, Greaves and Maley 2012, Beerenwinkel *et al.* 2015). Another disadvantage of coalescent models was that they assumed a well-mixed population, which may be incorrect for solid tumors (Beerenwinkel *et al.* 2015). Five methods iteratively applied a branching process to simulate either a clonal or cancer stem cell (CSC) model. Briefly, a clonal model was created by recursively generating subclones from a founder clone, and a CSC model by independently generating subclones from different founder clones (Shackleton *et al.* 2009, Ding *et al.* 2012). However, these methods did not allow for consideration of differential clone fitness, such as parameterization of each clone with different birth and death rates. Finally, two self-contained methods required a user-provided tree with alterations assigned to the tree nodes (Chu *et al.* 2017, Salcedo *et al.* 2020) (Table 1).

## 4 Simulation for multiple tumor samples is limited

Most self-contained methods reviewed were geared toward simulating tumor sequences for a single sample, with some offering the option to randomly generate sequences for multiple samples. By multiple samples, we refer to either multiple regions and/or multiple time points. One unique method enabled sample generation at specific points within a three-dimensional space (Yang *et al.* 2019). While many custom simulation methods were capable of simulating bulk DNA data across multiple samples, none of these methods, whether self-contained or custom, accounted for the varying selective pressures that subclones might experience in different spatial locations or over time. This points to a need for improvements to encompass both spatial and temporal patterns in multiple tumor samples.

## 5 Most methods lack targeted modalities

While whole-genome sequencing (WGS) databases, such as the Hartwig Medical Foundation WGS database, have been emerging and become available to researchers, whole-exome sequencing (WES) and gene panels are still widely used in the clinic for cost-effectiveness, and many tumor reconstruction algorithms were designed to recapture the tumor evolution using sequencing depth that can only be achieved by targeted panels (Priestley *et al.* 2019). Simulating tumor sequences from WES or gene panels would greatly aid the benchmarking of these targeted approaches. However, among the reviewed methods that generate raw reads, only two supported targeted sequencing, relying on genomic region coordinates in BED format and, for one method, a probe definition file (Yang *et al.* 2019, Srivatsa *et al.* 2023). These methods allow customization of DNA fragment and read lengths, as well as read depth, with one method using an external package for sequence read simulation based on these parameters (Yang *et al.* 2019). Given the lack of targeted modalities in most methods, future developments should focus on enhancing the inclusion of targeted sequencing methods like WES and gene panels in simulation models.

## 6 Error modeling for scDNAseq is deficient

Single-cell DNA sequencing identifies genetic information on a per-cell basis. In contrast to bulk sequencing, the mapping of reads to cells is observable, simplifying the process of subclonal reconstruction, but the results are error-prone. Errors include sample distortion, false negative errors (coverage and allelic dropout, allelic imbalance), false positive errors (amplification errors, doublets), and missing values. Briefly, sample distortion occurs during the cell isolation step, in which cells are selected for sequencing based on size, viability, or likelihood of entering the cell cycle (Gawad *et al.* 2016). Commonly used whole-genome amplification (WGA) methods prior to library construction, such as DOP-PCR, or MALBAC can introduce dropout, amplification errors, and substantially uneven coverage at different genomic locations (genome non-uniformity) (Fig. 1). Three of the simulation methods generated single-cell reads but did not incorporate any of these errors (Yang *et al.* 2019, Srivatsa *et al.* 2023). Two of the methods that generated reads incorporated genome non-uniformity (Mallory and Nakhleh 2022, Weiner and Bansal 2023). Many methods incorporated multiple sources of error, but they generated cell-by-mutation matrix profiles rather than actual sequence data (Table 1, Supplementary Table S1). Sequencing errors confound subclonal reconstruction algorithms, and modeling errors in scDNAseq data simulations is crucial for accurate benchmarking. Advances like DLP Plus may reduce scDNAseq errors, potentially lessening the need for simulating these errors in the future (Laks *et al.*, 2019). However, current error modeling in simulations must improve to match the technology's capabilities at present.

## 7 Few methods combined bulk and scDNAseq simulation

Bulk DNA sequencing broadly detects somatic alterations in tumors but fails to link these to specific cells, while scDNAseq provides cell-specific details but is error-prone and may overlook tumor heterogeneity due to limited cell sampling. Combining these methods can harness their strengths. Seven of the methods we reviewed support both bulk and scDNAseq simulations for the same sample (Table 1, Supplementary Table S1). We observed varied error-handling capabilities in these methods, with some addressing multiple error types and others focusing on specific aspects like genomic non-uniformity. Enhancing current methods to include both, especially with refined scDNAseq error modeling, would be beneficial.

## 8 Custom simulation methods predominate in self-benchmarking

We were surprised to discover that most subclonal reconstruction algorithms developed their own custom simulation methods for benchmarking purposes. Out of the 42 subclonal reconstruction algorithms (Supplementary Table S1), only two used the software packages listed in Table 1: FastClone used DREAM challenge simulated tumor sequence from BAMSurgeon (Ewing *et al.* 2015, Salcedo *et al.* 2020, Xiao *et al.* 2020), and CellPhy used CellCoal (Posada 2020, Kozlov *et al.* 2022). The rest of the algorithms generated custom-simulated data, which promoted less reproducible results and made unbiased comparisons between methods difficult.

## 9 Less reproducibility is seen in custom simulation methods

For scientific rigor, it is important that a benchmarking experiment can be reproduced by qualified researchers. Simulation reproducibility requires publishing either: (1) simulated sequence reads; (2) a high-level summary of the alterations; or (3) well-documented code that can generate sequence reads or a high-level summary, including necessary parameters. If the generation process includes randomization, a random seed must be included. If sequence reads are used, a well-documented, parameterized workflow for all steps of the analysis pipeline should be provided (genome alignment, germline and somatic alteration calling, as needed). Twelve of the subclonal reconstruction algorithms provided either the simulated sequence reads or the high-level summary of the data used in their benchmark experiments (Supplementary Table S1). The rest of the algorithms did not provide either type of benchmark data. Out of 42 algorithms, only 11 published their custom simulation code (Supplementary Table S1). The quality of documentation ranged from no instruction about how to run a simulator (El-Kebir 2018, Singer *et al.* 2018, Cmero *et al.* 2020, Myers *et al.* 2020, Kozlov *et al.* 2022, Markowska *et al.* 2022) to a simulator with a detailed usage guide (Ross and Markowetz 2016, Roth *et al.* 2016, Deveau *et al.* 2018, Zaccaria and Raphael 2020, Kang *et al.* 2022). Seven of the methods also published scripts or parameter files necessary to reproduce their benchmarking datasets (Ross and Markowetz 2016, Roth *et al.* 2016, El-Kebir 2018, Singer *et al.* 2018, Cmero *et al.* 2020, Kang *et al.* 2022, Kozlov *et al.* 2022). Overall, the greatest shortcomings in simulation methods, particularly the disparity between self-contained and custom methods, revolve around issues of reproducibility and bias. The findings highlight a need for more standardized, transparent, and replicable practices in the development and reporting of simulation methods to improve benchmarking in the field.

## 10 Enhancements are needed for emerging technologies

As new sequencing technologies are developed and applied to the analysis of tumor evolution, there are many opportunities for simulation methods to incorporate them. Long-read sequencers such as Sequel from Pacific Biosciences (PacBio) and MinION from Oxford Nanopore Technologies (ONT) can now produce reads about 100–1500 kb in length. This new generation of sequencers is enabling major improvements in phasing and SV calling, allowing the identification of subclones with allele-specific alterations and SVs that have not been detectable with short-read technologies (Amarasinghe *et al.* 2020, Logsdon *et al.* 2020, Maestri *et al.* 2020, Laufer *et al.* 2023). Simulation methods can incorporate existing long-read sequence generators to produce long reads for benchmarking (Shcherbina 2014, Escalona *et al.* 2016). Another exciting direction is the emergence of *in situ* sequencing methods, in which each sequenced read includes a unique identifier (UID) and barcode representing its spatial location (Zhao *et al.* 2022). Reads originating from a particular clone can be clustered and mapped to a spatial location on a tissue. This allows for observation of clone size, and when combined with single-cell transcriptomics, it is possible to identify the cell types and states present in each clone and its neighbors. *In situ* sequencing will play an important role in the next generation of subclonal reconstruction methods, and this will require new simulation methods that can generate realistic multi-modal *in situ* reads.

## 11 Application of simulation tools for other purposes

While we focus on simulation tools for tumor evolution, these tools have broader applications. For instance, to analyze mutational signatures across different tumors, one might simulate datasets using tools like BAMSurgeon or MosaicSim for bulk sequencing, and CellCoal for scDNAseq (Posada 2020, Salcedo *et al.* 2020, Srivatsa *et al.* 2023). SV discovery requires WGS, and one can use tools such as MosaicSim to test what percentage of SVs may be recovered through WES. These simulation tools may also aid in experimental design. For example, PSiTE may help to estimate how many samples one should collect to recover the maximum amount of tumor heterogeneity (Yang *et al.* 2019). Whether bulk sequencing or scDNAseq would be more suitable for analyzing a particular tumor type may be assessed by PSiTE and MosaicSim.

## 12 Recommendations

**Figure 2.**
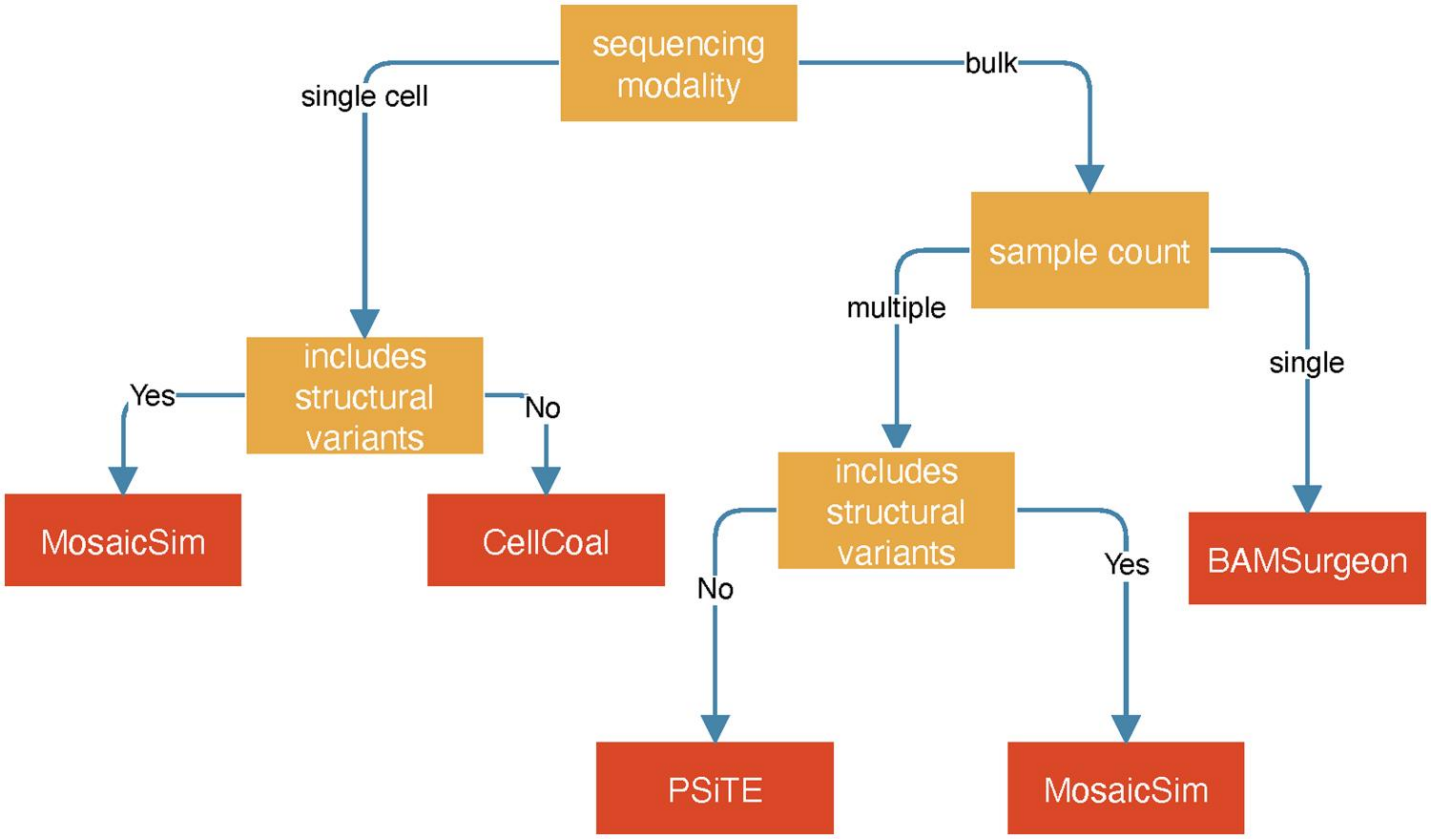
Decision tree for tool recommendation. Overall, we recommend BAMSurgeon if the user is interested in single-sample bulk sequencing and MosaicSim or PSiTE for multiple-sample bulk sequencing. CellCoal is a great tool for single-cell simulation.

- **Standardize simulation methods:** To enable consistent and reproducible benchmarking of subclonal reconstruction algorithms, the field needs widely accepted protocols for how simulation tools should be used, which will simplify the process of comparing different algorithms. This can accelerate advancements in the field, as it becomes clearer what methods are most effective.
- **Consider complexity:** Aim to have simulation methods reflect a broad range of genomic alterations found in tumors, including phased germline and somatic alterations, where possible.
- **Enhance targeted modality support:** Develop simulations that support targeted sequencing approaches like WES or gene panels to reflect current laboratory practices and clinical datasets.
- **Refine error modeling:** Improve error modeling in simulations, particularly for single-cell DNA sequencing data, to reflect the artifacts present in modern sequencing technologies.
- **Simulate multi-sample scenarios:** Extend simulation capabilities to cover multiple tumor samples and spatial locations, capturing intra-tumor heterogeneity and evolution.
- **Document and share:** Encourage comprehensive documentation and sharing of custom simulation codes and parameters to facilitate validation and replication of benchmarking results.
- **Cross-modality simulation:** Promote the development of methods capable of simulating both bulk and single-cell DNA sequencing data to leverage their combined strengths.
- **Community collaboration:** Unifying and enforcing standardized simulation tools can be challenging due to the constantly changing sequencing tools and the diverse input formats required by different subclonal reconstruction algorithms. Foster community-wide collaboration to refine and agree upon simulation standards and practices for the field. This ensures that the tools are not only used in a uniform manner but are also of a high standard.
- **Application of simulation methods:** We provide guidance for selecting the best available simulation tool under different scenarios (Fig. 2). For single-sample bulk sequencing, BAMSurgeon is recommended because it supports a wide range of somatic alteration types, introduces realistic and biologically plausible alterations, and has excellent documentation (Salcedo *et al.* 2020). PSiTE is ideal for multiple samples without structural variants, as it incorporates user-specified spatial information and is applicable to targeted sequencing platforms including gene panels (Yang *et al.* 2019). MosaicSim is suited for simulating multiple samples or single-cell sequencing with structural variants, as it uniquely supports these features (Srivatsa *et al.* 2023). We recommend CellCoal for single-cell sequencing without structural variants, because it has the most advanced features for handling sequencing errors in single-cell sequencing data without structural variants (Posada 2020).

## 13 Conclusion

Evaluation of subclonal reconstruction algorithms requires comparison with the ground truth about the evolutionary history of a neoplastic tumor. Currently, this ground truth cannot be measured experimentally. Longitudinal sampling of patient tumor tissue is limited by practical and ethical concerns, and technological limitations in detection sensitivity and resolution further complicate the process. While patient-derived xenografts (PDX) and organoids may be used in the future to better model tumor evolution, realistic simulated data is currently required for benchmarking. Here, we evaluated 51 simulation methods, considering their range of supported sequence alterations, sequencing modalities, assumptions about tumor evolution, reproducibility, and usability. We discovered that most simulation methods were developed in conjunction with a particular reconstruction algorithm. Because the types of somatic alterations and assumptions about the tumor evolutionary process differed substantially among the simulation methods, it would be difficult to get stable results when benchmarking subclonal reconstruction algorithms with different simulators. The results of most custom simulation methods could not be reproduced, because the code was either not published or not well documented. The diversity in methods and lack of reproducibility challenges effective benchmarking, highlighting the importance of developing more reliable and universally applicable simulation tools for tumor evolutionary studies.

## Supplementary Material

vbae094_Supplementary_Data
