# Supplementary material for: Assessing the merits: an opinion on the effectiveness of simulation techniques in tumor subclonal reconstruction
Source: Bioinform Adv. 2024 Jun 26;4(1):vbae094. doi: 10.1093/bioadv/vbae094 (PMC11213631; doi:10.1093/bioadv/vbae094)
Supplement: vbae094_Supplementary_Data [file vbae094_supplementary_data.pdf]

Supplementary Table S1. Simulation methods used to benchmark 42 subclonal reconstruction algorithms. Algorithms that used a self-contained method from Table 1 are indicated in bold type. Forty methods were self-benchmarked by a custom simulation method. **G**=germline, **S**=somatic, **GS**=germline and somatic, **SNV**=single nucleotide variant, **Indel**=small insertion/deletion, **CNA**=copy number alteration, **SV**=structural variant, **AP**=aneuploidy, **MS**=mutation signature. Details of supported sequencing modalities are **OT**=output type(🧬=raw reads, 📕=summary data such as reads counts for SNVs or segmented read depth for CNAs). Bulk sequencing features are **SE**=sequencing error, **BQ**=base quality, **GC**=GC bias, **WES**=whole-exome sequencing, **GP**=gene panel. scDNAseq errors are **FN**=false negative(such as allelic dropout and allelic imbalance), **FP**=false positives(such as amplification error and doublets), **MV**=missing value (result of non-uniform genome coverage), **SD**=sample distortion. **MSS**=multiple sample simulated , **Data**=simulated data used for benchmarking, and **Code**=simulation code. 📖=code includes detailed documentation, ⭐=parameters or shell scripts to reproduce simulation. Tree types are: **BP**=branching process, **CA**=coalescent, **RT**=random topology, or **EX**=exhaustive for fixed N, where N is the number of nodes on the tree. *=pre-computed trees were provided, and the number of trees is indicated.

Reconstruction Algorithm	Custom Simulation Methods																					
	Genome alterations						Sequencing modalities											Tree	MSS	Data	Code	Reference
							bulk DNA sequencing						scDNAseq									
	SNV	Indel	CNA	SV	AP	MS	OT	SE	BQ	GC	WES	GP	OT	FP	FN	MV	SD					
**CellPhy**	S	-	-	-	-	-	-	-	-	-	-	-	📕	✓	✓	✓	-	CA	-	✓	✓⭐	Kozlov et al., 2022
**FastClone**	GS	GS	GS	GS	✓	✓	🧬	✓	✓	✓	-	-	-	-	-	-	-	8*	-	✓	-	Xiao et al., 2020
SVClone	G	-	-	S	-	-	🧬	✓	✓	✓	-	-	-	-	-	-	-	1*	-	✓	✓⭐	Cmero et al., 2020
SPhyR	S	-	-	-	-	-	-	-	-	-	-	-	📕	✓	✓	-	-	RT	-	✓	✓⭐	El-Kebir, 2018
HATCHet	G	-	S	-	✓	-	🧬	✓	✓	✓	-	-	-	-	-	-	-	RT	✓	✓	✓📖	Zaccaria and Raphael, 2020
CNTMD	-	-	S	-	-	-	📕	-	-	-	-	-	-	-	-	-	-	RT	✓	✓	-	Zaccaria et al., 2017
DEVOLUTION	-	-	S	-	-	-	📕	-	-	-	-	-	-	-	-	-	-	BP	✓	✓	-	Andersson et al., 2021
SPRUCE	S	-	S	-	-	-	📕	-	-	-	-	-	-	-	-	-	-	RT	✓	✓	-	El-Kebir et al., 2016
PhyloWGS	S	-	S	-	-	-	📕	-	-	-	-	-	-	-	-	-	-	RT	-	✓	-	Deshwar et al., 2015
SubMARine	S	-	S	-	-	-	📕	-	-	-	-	-	-	-	-	-	-	RT	✓	✓	-	Sundermann et al., 2021
TUSV-ext	S	-	S	S	-	-	📕	-	-	-	-	-	-	-	-	-	-	1*	✓	✓	-	Fu et al., 2022
ddClone	S	-	S	-	-	-	📕	-	-	-	-	-	📕	✓	✓	-	✓	RT	-	✓	-	Salehi et al., 2017
OncoNEM	S	-	-	-	-	-	-	-	-	-	-	-	📕	✓	✓	✓	-	RT	-	-	✓📖⭐	Ross and Markowetz, 2016
SCG	S	-	-	-	-	-	-	-	-	-	-	-	📕	✓	✓	✓	-	RT	-	-	✓📖⭐	Roth et al., 2016
SIEVE	GS	S	S	-	-	-	-	-	-	-	-	-	📕	✓	✓	✓	-	BP	-	-	✓📖⭐	Kang et al., 2022
SCIΦ	S	-	S	-	-	-	-	-	-	-	-	-	📕	✓	✓	✓	-	RT	-	-	✓⭐	Singer et al., 2018

QuantumClone	S	-	-	-	✓	-	📕	-	-	-	-	-	-	-	-	-	-	RT	✓	-	✓📖	Deveau et al., 2018
CONET	-	-	S	-	-	-	-	-	-	-	-	-	📕	-	-	-	-	RT	-	-	✓	Markowska et al., 2022
SBMClone	S	-	-	-	-	-	-	-	-	-	-	-	📕	-	-	✓	-	2*	-	-	✓	Myers et al., 2020
CITUP	S	-	-	-	-	-	📕	-	-	-	-	-	-	-	-	-	-	RT	✓	-	-	Malikic et al., 2015
Rec-BTP	S	-	-	-	-	-	📕	-	-	-	-	-	-	-	-	-	-	EX	-	-	-	Hajirasouliha et al., 2014
PhyloSub	S	-	-	-	-	-	📕	-	-	-	-	-	-	-	-	-	-	1*	-	-	-	Jiao et al., 2014
AncesTree	S	-	-	-	-	-	📕	-	-	-	-	-	-	-	-	-	-	RT	✓	-	-	El-Kebir et al., 2015
TrAp	S	-	-	-	-	-	📕	-	-	-	-	-	-	-	-	-	-	RT	-	-	-	Strino et al., 2013
ClonosGP	S	-	-	-	-	-	📕	-	-	-	-	-	-	-	-	-	-	RT	✓	-	-	Vavoulis et al., 2021
SCHISM	S	-	-	-	-	-	📕	-	-	-	-	-	-	-	-	-	-	EX	✓	-	-	Niknafs et al., 2015
PICTograph	S	-	-	-	-	-	📕	-	-	-	-	-	-	-	-	-	-	EX	✓	-	-	Zheng et al., 2022
THetA	-	-	S	-	-	-	📕	-	-	-	-	-	-	-	-	-	-	1*	-	-	-	Oesper et al., 2013
LICHeE	S	-	S	-	-	-	📕	-	-	-	-	-	-	-	-	-	-	BP	✓	-	-	Popic et al., 2015
PyClone	S	-	S	-	-	-	📕	-	-	-	-	-	-	-	-	-	-	1*	-	-	-	Roth et al., 2014
DeCiFer	S	-	S	-	✓	-	📕	-	-	-	-	-	-	-	-	-	-	RT	✓	-	-	Satas et al., 2021
Canopy	S	-	S	-	-	-	📕	-	-	-	-	-	-	-	-	-	-	4*	✓	-	-	Jiang et al., 2016
cloneHD	GS	-	S	-	-	-	📕	-	-	-	-	-	-	-	-	-	-	1*	✓	-	-	Fischer et al., 2014
MELTOS	S	-	S	S	-	-	🧬	✓	✓	✓	-	-	-	-	-	-	-	BP	✓	-	-	Ricketts et al., 2020
BnpC	S	-	-	-	-	-	-	-	-	-	-	-	📕	✓	✓	✓	-	RT	-	-	-	Borgsmüller et al., 2020
SCITE	S	-	-	-	-	-	-	-	-	-	-	-	📕	✓	✓	✓	-	RT	-	-	-	Jahn et al., 2016
Ginkgo	-	-	S	-	-	-	-	-	-	-	-	-	🧬	-	-	-	-	1*	-	-	-	Garvin et al., 2015
SiFit	S	S	S	-	-	-	-	-	-	-	-	-	📕	✓	✓	✓	-	RT	-	-	-	Zafar et al., 2017
SiCloneFit	S	-	S	-	-	-	-	-	-	-	-	-	📕	✓	✓	✓	-	RT	-	-	-	Zafar et al., 2019
Conifer	S	-	S	-	-	-	📕	-	-	-	-	-	📕	✓	✓	✓	✓	RT	-	-	-	Baghaarabani et al., 2021
PhISCS	S	-	-	-	-	-	📕	-	-	-	-	-	📕	✓	✓	✓	-	RT	-	-	-	Malikic et al., 2019
B-SCITE	S	-	S	-	-	-	📕	-	-	-	-	-	📕	✓	✓	✓	✓	RT	-	-	-	Malikic et al., 2019
